# Supplementary material for: Percolation on feature-enriched interconnected systems
Source: Nat Commun. 2021 Apr 30;12:2478. doi: 10.1038/s41467-021-22721-z (PMC8087700; doi:10.1038/s41467-021-22721-z)
Supplement: Supplementary file 1 — Supplementary Information [file 41467_2021_22721_MOESM1_ESM.pdf]

Supplementary Information for *Percolation on feature-enriched interconnected systems*

Oriol Artime[1,*] and Manlio De Domenico[1,]**

[1]Center for Information and Communication Technology, Fondazione Bruno Kessler, Via Sommarive 18, 38123 Povo (TN), Italy
[*]oartime@fbk.eu
[**]mdedomenico@fbk.eu

Supplementary Note 1: Uncorrelated $P(k,F)$ in Erdős-Rényi networks

We construct networks with the degree distribution $p_k = e^{-c}c^k/k!$, and independently we draw a feature value for each node from the distribution $p(F) = (\alpha-1)F^{-\alpha}$, with $F \geq 1$ and $\alpha > 1$. Recall that k is discrete and F is continuous, although it is straightforward to repeat the calculations assuming F discrete as well. The considered occupation probability is $\phi_F = \theta(-(F-F_0))$.

The joint distribution $P(k,F)$ is separable, thus the k and F contributions in the generating functions can be computed separately. On the one hand we have

$$\int \mathrm{d}F P(F)\phi_F = 1 - F_0^{1-\alpha}. \tag{1}$$

On the other hand,

$$\sum_{k=0}^{\infty} p_k u^k = e^{-c}\sum_{k=0}^{\infty}\frac{c^k}{k!}u^k = e^{c(u-1)} \tag{2}$$

and

$$\sum_{k=0}^{\infty} q_k u^k = \frac{1}{\langle k\rangle}\sum_{k=0}^{\infty}(k+1)p_{k+1}u^k = \frac{e^{-c}}{c}\sum_{k=0}^{\infty}(k+1)\frac{c^{k+1}}{(k+1)!}u^k = e^{c(u-1)}. \tag{3}$$

Combining both results, we get

$$g_0(u) = \left(1-F_0^{1-\alpha}\right)e^{c(u-1)} \tag{4}$$

$$g_1(u) = \left(1-F_0^{1-\alpha}\right)e^{c(u-1)}. \tag{5}$$

The criticality condition $1 = \partial_z g_1(z)|_{z=1}$ yields

$$c_c = \frac{1}{1-F_0^{1-\alpha}} \tag{6}$$

$$\alpha_c = 1 - \frac{\log\left(1-\frac{1}{c}\right)}{\log(F_0)}. \tag{7}$$

Results comparing the analytical curves and the simulations are shown in 1, finding a good agreement between theory and simulations.

Supplementary Note 2: Uncorrelated $P(k,F)$ in scale-free networks

We consider networks with degree distribution $p_k = k^{-\gamma}/\zeta(\gamma)$, $k \geq 1$. The feature distribution is the same as before. Noting that $\langle k\rangle = \zeta(\gamma-1)/\zeta(\gamma)$, where $\zeta(\gamma)$ is the zeta Riemann function, the topological part of the generating functions read

$$\sum_{k=1}^{\infty} p_k u^k = \frac{1}{\zeta(\gamma)}\sum_{k=1}^{\infty}k^{-\gamma}u^k = \mathrm{Li}_\gamma(u)/\zeta(\gamma), \tag{8}$$

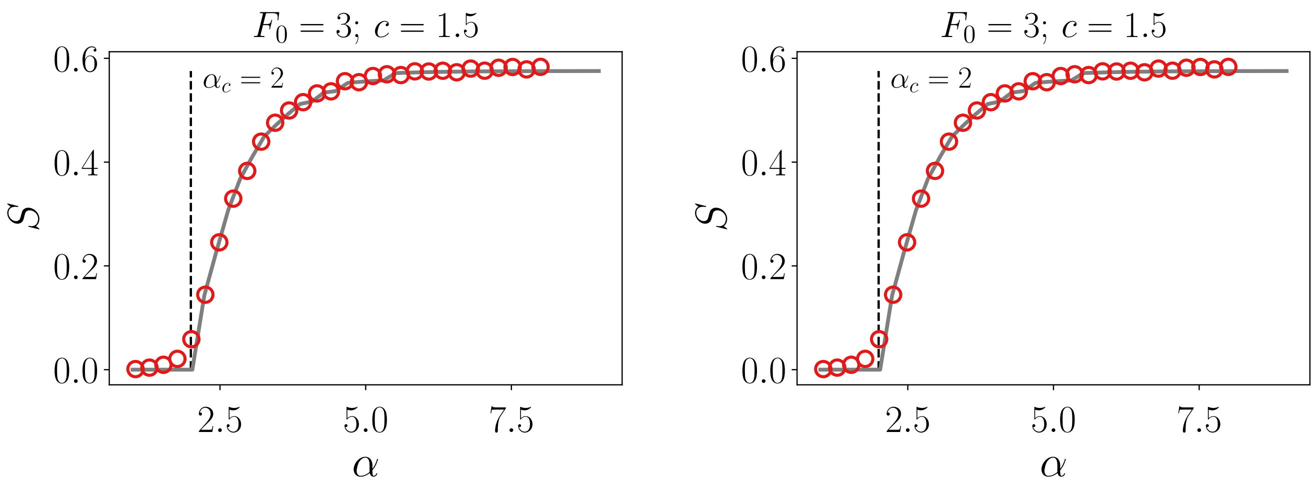

Supplementary Figure 1. Feature-based percolation in Erdős-Rényi networks with uncorrelated degree and feature. On the left, the size of giant component as a function of the network parameter. On the right, the size of giant component as a function of the feature parameter. Network size is $N = 2000$, averaged over 100 realizations.

and

$$\sum_{k=0}^{\infty} q_k u^k = \frac{1}{\langle k\rangle}\frac{\partial}{\partial u}\sum_{m=1}^{\infty} p_m u^m = \frac{\mathrm{Li}_{\gamma-1}(u)}{u\zeta(\gamma-1)}. \tag{9}$$

where $\mathrm{Li}_{\gamma}(u)$ is the polylogarythm function. Thus, we have

$$g_0(u) = \left(1 - F_0^{1-\alpha}\right)\frac{\mathrm{Li}_{\gamma}(u)}{\zeta(\gamma)} \tag{10}$$

$$g_1(u) = \left(1 - F_0^{1-\alpha}\right)\frac{\mathrm{Li}_{\gamma-1}(u)}{u\zeta(\gamma-1)}. \tag{11}$$

The condition for the critical point is given by

$$\zeta(\gamma-1) = (1 - F_0^{1-\alpha})\left[\mathrm{Li}_{\gamma-2}(1) - \mathrm{Li}_{\gamma-1}(1)\right]. \tag{12}$$

Isolating the exponent of feature distribution we get

$$\alpha_c = 1 - \frac{1}{\log(F_0)}\log\left(1 - \frac{\zeta(\gamma-1)}{\mathrm{Li}_{\gamma-2}(1) - \mathrm{Li}_{\gamma-1}(1)}\right). \tag{13}$$

For the discussion of the behavior of α_c we provide in 2 the plots of the special functions involved in the condition of criticality. Note that $\mathrm{Li}_{\lambda}(1)$ diverges when $\lambda = 1$ and 2, and $\zeta(\lambda)$ does so when $\lambda = 1$. Therefore, at these points the critical point does not exist and S is finite for any value of α, as far as $\alpha > 1$. What does occur in the range $\lambda \in (1,2)$? It turns out that in this region $\zeta(\lambda) > \mathrm{Li}_{\lambda-1}(1) - \mathrm{Li}_{\lambda}(1)$, condition that makes the argument of the logarithm in Supplementary Equation (13) negative. Setting $\lambda = \gamma - 1$, we conclude then that the order parameter does not have a finite α_c in $\gamma \in [2,3]$.

Following similar argument we can shed light on the critical behavior feature-based percolation in scale-free networks with degree exponent $\gamma \in (1,2)$ and $\gamma > 3$. In the former, one finds that $\zeta(\gamma-1) < 0$ and $\mathrm{Li}_{\gamma-2}(1) - \mathrm{Li}_{\gamma-1}(1) > 0$. Since $1 - F_0^{1-\alpha}$ is always positive, the criticality condition is not hold, therefore there is no valid α_c. In the latter case, there is a small region, up to $\gamma \approx 3.478$, for which $\zeta(\gamma-1) > \mathrm{Li}_{\gamma-2}(1) - \mathrm{Li}_{\gamma-1}(1)$, implying a finite critical value α_c. For larger values of γ, the critical point does not exist.

Results comparing the analytical curves and the simulations are shown in 2. The agreement between theory and simulations is good. Let us remark the surprising effect that feature-enriched percolation has on scale-free networks, that is, for graphs with very broad degree distribution ($\gamma \leq 3$) and for any value of the feature threshold F_0 and feature exponent α, their giant component is always macroscopic. Put otherwise, these graphs are ultra-resilient to feature-based attacks. For the same type of attacks –equal F_0– and same α, vulnerability increases with the exponent of the degree distribution, since we find a region where the network can be completely dismantled. However, α_c does not increase indefinitely with the degree exponent λ, but from a certain point $\gamma \approx 3.478$, independent of F_0, we are back to the situation where α_c does not exist.

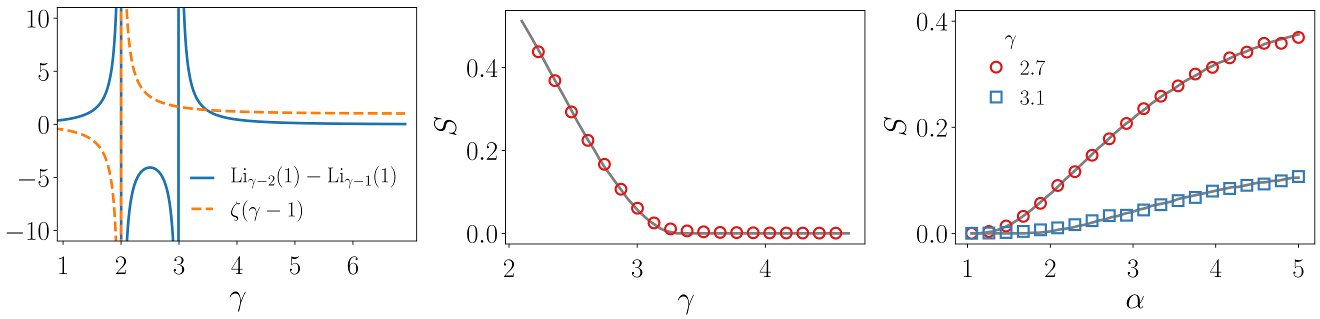

Supplementary Figure 2. Feature-based percolation in scale-free networks. On the left, we plot the behavior of the special functions that appear on both sides of the equation of the criticality condition (Supplementary Equation (12)). In the middle, we show the size of the giant component S as a function of the parameter related to the topology, the exponent of the degree distribution. We fix the other parameters to $F_0 = 2$ and $\alpha = 2.75$ On the right, it is displayed S as a function of the parameter related to the feature, the exponent of the power-law feature distribution. Two curves are shown, one for which α_c does not exist in the valid domain of α, even though $S(\alpha) \to 0$ for $\alpha \to 1$, and other for which α_c exists. $F_0 = 2$ here as well. Network size is $N = 20000$ and each point is averaged over 100 realizations.

Supplementary Note 3: Critical exponents in the independent case

Here we sketch the steps to obtain the critical exponents associated to the size of the giant component[1]. The starting point is main text's Equation (5). We change $u = 1-\varepsilon$ and $a = a_c + \delta$ and expand for $\varepsilon \to 0$ and $\delta \to 0$, to get

$$-\varepsilon = \left(1 - F_0^{1-\alpha}\right)\left[\frac{2a_c}{a_c-1}\varepsilon + \frac{3a_c^2}{(a_c-1)^2}\varepsilon^2 - \frac{2}{(a_c-1)^2}\varepsilon\delta + \mathscr{O}(\varepsilon\delta^2) + \mathscr{O}(\varepsilon^2\delta)\right]. \tag{14}$$

Dividing both sides by ε and noticing that

$$-1 = \left(1 - F_0^{1-\alpha}\right)\frac{2a_c}{a_c-1}, \tag{15}$$

we obtain that, at first order, $\varepsilon \sim 2/3a_c^{-2}\delta$. Now we proceed as before, substituting $u = 1-\varepsilon$ and $a = a_c + \delta$ in main text's Equation (1) and Taylor expanding around $\varepsilon \to 0$ and $\delta \to 0$:

$$S(a) = \left(1 - F_0^{1-\alpha}\right)\left[-\frac{a_c}{a_c-1}\varepsilon + \frac{\varepsilon\delta}{(a_c-1)^2} + \mathscr{O}(\varepsilon^2) + \mathscr{O}(\varepsilon\delta^2)\right]. \tag{16}$$

Keeping first-order terms and using the linear relation between ε and δ we obtain that

$$S(a) = \frac{\left(3 - 2F_0^{1-\alpha}\right)^2}{3}\delta \sim (a - a_c). \tag{17}$$

Therefore, we conclude that $\beta_a = 1$. The procedure to obtain β_α is exactly the same, although it becomes a bit more tedious because the variable α appears as an exponent. Applying carefully the same steps, we arrive at $\beta_\alpha = 1$.

Supplementary Note 4: Collapses in uncorrelated $P(k,F)$

We haven shown analytically and numerically that the geometric network $p_k = (1-a)a^k$ with uncorrelated feature distribution $p(F) = (\alpha-1)F^{-\alpha}$ have the same critical exponents as those in standard mean-field percolation. To support this result in an alternative way, we show that finite-size collapses correctly overlap when using the predicted exponents $\beta_a = \beta_\alpha = 1$ and $\overline{\nu}_a = \overline{\nu}_\alpha = 3$, see 3.

Supplementary Note 5: Limit $\alpha \to 1$ for the positively correlated model

Here we show why $S(\alpha) \to 0$ when $\alpha \to 1$ for the network model of main text's Equation (10) when nodes with feature larger than F_0 are removed. Starting from the generating functions, main text's Equations (12), the probability u that a node does not

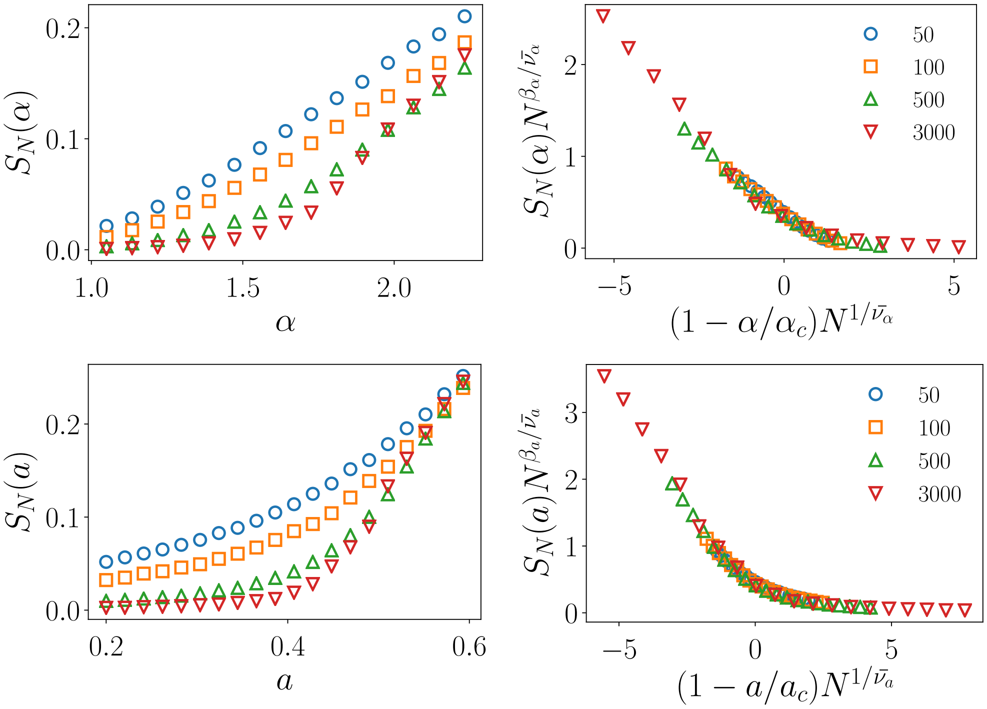

Supplementary Figure 3. Data collapses for the uncorrelated network model studied in the main text. S_N standard for the size of the largest component in simulations with networks of size N. On the top we show the analysis as a function of the feature parameter α and below as a function of the topological parameter a. In all simulations is used $F_0 = 3$ and averages are computed between over 5000 realizations (smallest system sizes) and over 100 (largest system sizes).

belong to the giant component via one neighbor is given by the transcendent equation

$$u = 1 - \frac{1}{\zeta(\alpha) - \zeta(\alpha+1)}[\zeta(\alpha,2) - \zeta(\alpha+1,2) - \zeta(\alpha,F_0+1) + F_0\zeta(\alpha+1,F_0+1) \\ - \Phi(u,\alpha,2) + \Phi(u,\alpha+1,2) + \Phi(u,\alpha,F_0+1) - F_0\Phi(u,\alpha+1,F_0+1)]. \quad (18)$$

Both $\zeta(\alpha)$ and $\zeta(\alpha, F_0+1)$ diverge for $\alpha \to 1$, so it seems that we encounter an indetermination. Luckily, they diverge at the same pace, so $\lim_{\alpha\to 1} \zeta(\alpha,F_0+1)/\zeta(\alpha) = 1$. Applying the limit to the entire equation we obtain that $u = 1$. By definition, S is vanishes for $u = 0$, but one can take the appropriate limits to the expression of the giant component to see that the approach is continuous. Taking $u \to 1$ and $\alpha \to 1$ in

$$S(u,\alpha,F_0) = 1 - \frac{\zeta(\alpha+1,F_0+1) + \Phi(u,\alpha+1,2) - u\Phi(u,\alpha+1,F_0+1)}{\zeta(\alpha+1) - 1} \quad (19)$$

we obtain 0, as shown in 3.

Supplementary Note 6: Universality classes for correlated $P(k,F)$

We proceed in this section to numerically check whether the correlated and anticorrelated models scenarios proposed in main text's Equations (10) and (14) belong to the mean-field percolation class, as it occurs with the uncorrelated case, main text's Equations (6).

Let us first focus on the positively correlated case. We show in 4(a) that the theoretical order parameter approaches linearly to the critical point, hence the critical exponent $\beta = 1$. Simulating the percolation process for different sizes we obtain S_N (4(b)), which neatly overlap when applying the finite-size scaling (4(c)) with $\beta = 1$ and $\bar{\nu}_\alpha = 3$. Therefore we conclude that the type of positive degree-feature correlations studied in the main text does not change the mean-field critical properties. Repeating the same procedure for the randomized version of the model, we confirm that it also belongs to the mean-field percolation class, see the bottom row of 4.

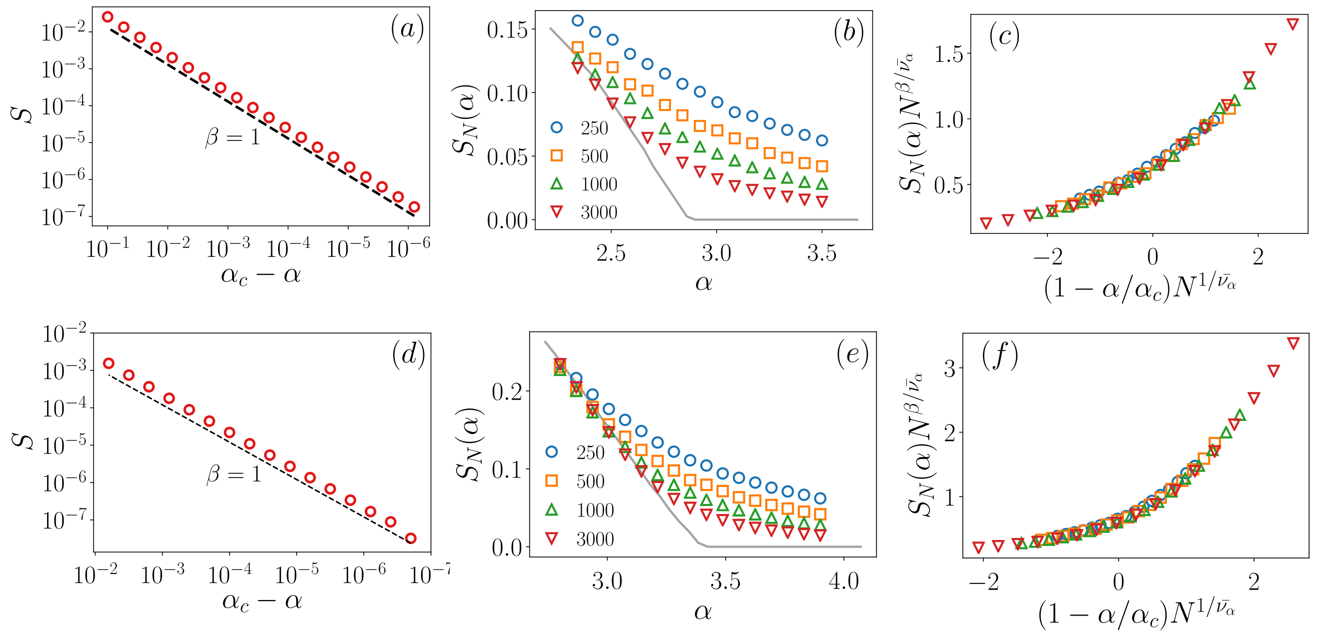

Supplementary Figure 4. Exploring the critical exponents of the positively correlated case. In (a), points correspond to the numerical solution of the theoretical order parameter, as a function of the distance to the critical point. To guide the eye, the dashed line $(\alpha_c - \alpha)^\beta$ with $\beta = 1$. In (b), results for the size of the largest connected component from simulations with different network sizes, indicated in the legend. Each point is computed by averaging at least 1000 realizations. The solid line is the theoretical solution. In (c), same data applying the finite-size scaling with $\beta = 1$ and $\overline{\nu}_\alpha = 3$. In (d), (e) and (f) we show the same analysis for the randomized version of the model.

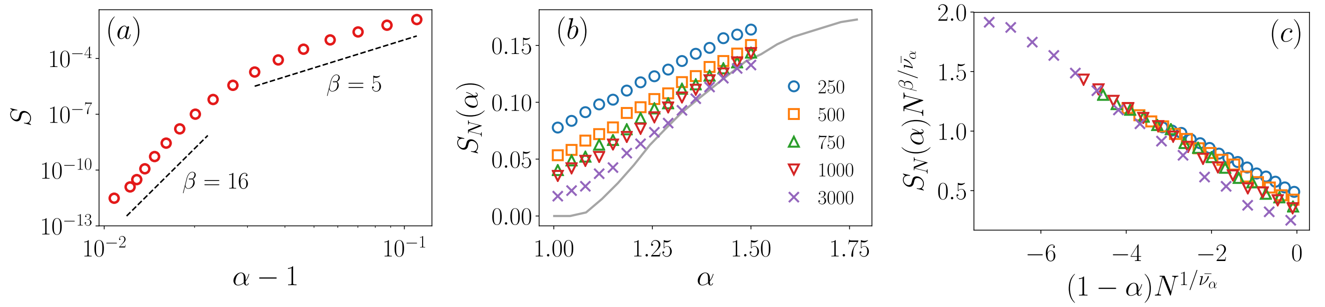

Supplementary Figure 5. Exploring the possible critical behavior the positively correlated case close to $\alpha = 1$. In (a), points correspond to the numerical solution of the theoretical order parameter, as a function of the distance to the point of interest. To guide the eye, two power laws are incorporated, the dashed line $(\alpha - 1)^\beta$ with $\beta = 1$. In (b), results for the size of the largest connected component from simulations with different network sizes, indicated in the legend. Each point is computed by averaging at least 1000 realizations. The solid line is the theoretical solution. In (c), same data applying the finite-size scaling with $\beta = 1$ and $\overline{\nu}_\alpha = 3$.

We see that positive correlations make $S \to 0$ as $\alpha \to 1$ (3(a)). However, $\alpha = 1$ is outside the range of valid values of the control parameter. It is instructive to study what happens in the vicinity of that value in order to see if we find any signature of criticality. This is explored in 5. Studying the behavior of theoretical solution, we observe that not only there is no clear power law decay (5(a)), hence not verifying the scaling hypothesis, but also the decay occurs very abruptly. Proceeding similarly as before, we simulate the process for small-to-intermediate system sizes (5(b)) and apply the scaling transformation. In 5(c) we confirm that the curves do not overlap, if using the mean-field exponents. The collapse also fails if we employ the greater β values suggested in 5, for any value of $\overline{\nu}_\alpha$, see the Supplementary Movies 1 and 2. All these results offer strong evidence that there is no critical behavior in the vicinity of $\alpha = 1$ even though $S \to 0$.

To close this section we study the universality class of the negatively correlated model, proceeding as before. The first

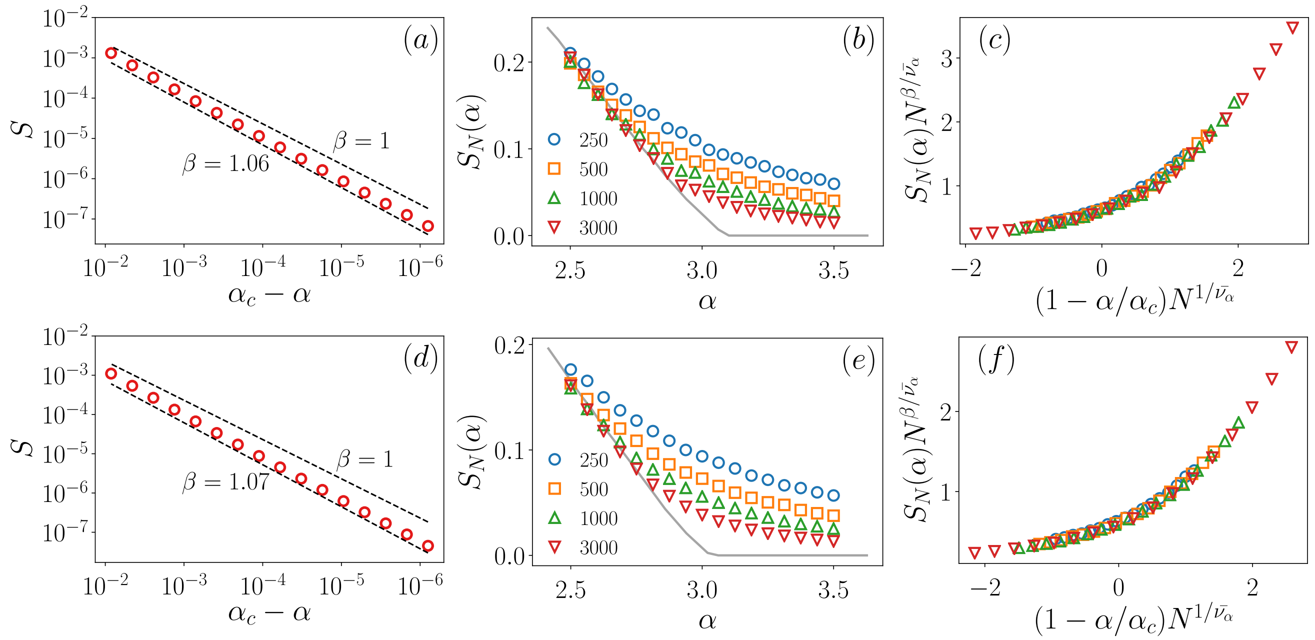

Supplementary Figure 6. Critical exponents in the negatively correlated model. In (a), points correspond to the numerical solution of the theoretical order parameter, as a function of the distance to the critical point. Two power laws are shown, one with the mean-field exponent, which deviates from the theoretical solution, and another that fits better the data. In (b), results for the size of the largest connected component from simulations with different network sizes, indicated in the legend. Each point is computed by averaging 4000 realizations. The solid line is the theoretical solution. In (c), same data applying the finite-size scaling with the exponent β obtained in (a) and $\overline{\nu}_\alpha = 3$. In (d), (e) and (f), same plots for the randomized case.

observation is that, for both the anticorrelated and randomized cases, the exponent β is slightly higher than its mean-field value 1 (6(a) and (d)). The simulated data (6(b) and (e)) overlaps well if using the value of β obtained from the theoretical solution and employing $\overline{\nu}_\alpha = 3$. We cannot discard, however, that the true value of the exponent of the correlation length $\overline{\nu}_\alpha$ is different but close to its mean-field value 3. This requires further analysis, such as obtaining the expression of a quantity from which we can extract analytically and independently other critical exponents, e.g., the mean cluster size, and from there apply the scaling relations between critical exponents. It also remains open the question why positive correlations do not change the universality class but negative correlations do.

Supplementary Note 7: Joint degree-feature probability function for Random Geometric Graphs

Here we show how to compute the proper joint distribution $P(k, d_{min})$, where the feature d_{min} is taken as the distance between a node and its closest neighbor. The steps to follow are conceptually very simple: first we compute the degree distribution p_k and the conditional feature-degree distribution $P(d_{min}|k)$ by employing purely geometrical arguments and basic probabilistic relations, and then, by definition, we readily obtain the joint distribution $P(k, d_{min}) = P(d_{min}|k)p_k$.

Let us start with the degree distribution. In a two-dimensional random geometric graph of neighborhood radius r, with periodic boundary conditions, the number of nodes k distance r from a randomly chosen node follows the binomial distribution with the area of circle of interaction as a parameter, i.e.,

$$p_k = \binom{N-1}{k} (\pi r^2)^k (1-\pi r^2)^{N-1-k}. \tag{20}$$

Its mean degree is $\langle k \rangle = (N-1)\pi r^2$, as expected.

The computation of $P(d_{min}|k)$ is much trickier. The probability that a randomly chosen neighbor is located at a distance between d and $d + \mathrm{d}d$ from a node is given by

$$P(d) = \frac{2d}{r^2}. \tag{21}$$

Assume we have k neighbors randomly distributed following Supplementary Equation (21). Then the probability $P(d_{min}|k)$ that the closest neighbor is at distance d_{min} can be obtained by employing the fact that $C(d_{min}|k) = 1-(1-C(d_{min}))^k$, where $C(d_{min}|k)$ and $C(d_{min})$ are the cumulative functions of the conditional probability $P(d_{min}|k)$ and of $P(d_{min})$. We immediately obtain that

$$C(d_{min}|k) = 1-\left(1-\frac{d_{min}^2}{r^2}\right)^k, \tag{22}$$

which leads to

$$P(d_{min}|k) = \frac{2d_{min}}{r^2}k\left(1-\frac{d_{min}}{r^2}\right)^{k-1}. \tag{23}$$

In principle, the product of Supplementary Equation (20) and Equation (23) was our goal. However, note that Equation (23) is well-normalized to unity for all degrees but $k=0$. This is because these nodes, that occur with a finite probability $p_0 = (1-\pi r^2)^{N-1}$, do not have a minimum distance d_{min} to the closest neighbor because they do not have neighbors. We overcome this problem by manually assigning $d_{min}=0$ when $k=0$, that will not affect the percolation properties since $k=0$ never contribute to the giant component, but will modify the distributions making it correctly normalizable to unity.

We have now two contributions in the probability distribution, one with discrete support at 0 and the other in a continuous support $[0,r]$. The probability function is given by $P(d_{min}) = \delta(d_{min})p_{k=0} + \sum_{k=1}^{N-1} P(d_{min}|k)p_k$, where $\delta(\cdot)$ is Dirac's delta. The correctly normalized distributions are then

$$\begin{aligned}
P(d_{min}|k) &= \delta_{k,0}\delta(d_{min}) + \left(1-\delta_{k,0}\right)\left[\frac{2d_{min}}{r^2}k\left(1-\frac{d_{min}}{r^2}\right)^{k-1}\right],\\
C(d_{min}|k) &= \delta_{k,0} + \left(1-\delta_{k,0}\right)\left[1-\left(1-\frac{d_{min}^2}{r^2}\right)^k\right],\\
P(d_{min}) &= \delta(d_{min})\left(1-\pi r^2\right)^{N-1} + 2\pi(N-1)d_{min}\left(1-\pi d_{min}^2\right)^{N-2},\\
C(d_{min}) &= 1-\left(1-\pi d_{min}^2\right)^{N-1} + \left(1-\pi r^2\right)^{N-1},
\end{aligned} \tag{24}$$

where we have used, moreover, the Kronecker delta to differentiate the cases $k=0$ and $k>0$. We finally can write the correct joint probability distribution

$$P(k,d_{min}) = \delta_{k,0}\delta(d_{min})(1-\pi r^2)^{N-1} + \left(1-\delta_{k,0}\right)\binom{N-1}{k}(\pi r^2)^k(1-\pi r^2)^{N-1-k}\frac{2d_{min}}{r^2}k\left(1-\frac{d_{min}}{r^2}\right)^{k-1}. \tag{25}$$

Notice that the joint distribution Supplementary Equation (25) is indeed well normalized to unity, and that the term accompanied with the δ-functions is necessary to be so.

Supplementary Note 8: Output functions of the Bayesian Machine Scientist

Here we write down the functions given by the BMS that have been used to compute the joint degree-feature distribution when analysing the dynamical models on the real topologies, main text's Equation (21) . For the mutualistic dynamics, 6(a), the mean value is $\mu_F(k) = a_{11} + a_{12}k^{a_{13}}$ and height of the probability peaks is $h(k) = a_{21}\sin\left(a_{22}k^{a_{23}}\right)$, with $a_{11} = 0.0019$, $a_{12} = 0.0022$, $a_{13} = 0.9996$, $a_{21} = 0.0193$, $a_{22} = 1.12\cdot 10^{-38}$ and $a_{23} = 14.4823$. For the population dynamics, 6(b), we obtain $\mu_F(k) = b_{11}\log\left(2k + b_{12}k^2\right)^2$ and $h(k) = b_{21} + b_{22}k + k^{b_{23}}$ with $b_{11} = 0.0114$, $b_{12} = 0.0158$, $b_{21} = 0.0024$, $b_{22} = -2.87\cdot 10^6$ and $b_{23} = -3.5861$. Finally, for the biochemical dynamics, 6(c), $\mu_F(k) = c_{11} + c_{12}\tan(k) + k^{c_{13}}$, $\sigma_F(k) = c_{21}^{k^{c_{22}} + \tan(k)/c_{23}} - \mu_F(k)$ and $h(k) = (c_{31} + c_{31}/(c_{32}+k))/k$ with $c_{11} = -0.109$, $c_{12} = -0.002$, $c_{13} = -0.4088$, $c_{21} = 0.795$, $c_{22} = 0.6029$, $c_{23} = 49.7377$, $c_{31} = 0.15$ and $c_{32} = -13.087$. In (a) and (b), since the standard deviation $\sigma_F(k)$ is approximately constant, instead of finding it by means of the BMS we set manually the value of 0.01. The range of degrees where these functions are defined are $[290,452]$ for the mutualistic dynamics, $[16,826]$ for the population dynamics and $[1,271]$ for the biochemical dynamics.

Notice that some of the values of the constants given by the BMS are considerably small or large. Owing to the stochastic nature of the algorithm, the values and the functions shown above are not always stable under the repetition of the experiment. In spite of this, even if the values of the parameters and the functions cannot be guaranteed to be the same from realization to realization, the approach of using the BMS to feature-enriched percolation is still valid because we are just looking for an approximate $P(k,F)$ and the outputs will capture very well the trends in the data fed to the algorithm.

Supplementary References

1. Cohen, R., Ben-Avraham, D. & Havlin, S. Percolation critical exponents in scale-free networks. *Phys. Rev. E* **66**, 036113 (2002).
