# Supplementary material for: Percolation on feature-enriched interconnected systems
Source: Nat Commun. 2021 Apr 30;12:2478. doi: 10.1038/s41467-021-22721-z (PMC8087700; doi:10.1038/s41467-021-22721-z)
Supplement: Supplementary file 3 — Description of Additional Supplementary Files [file 41467_2021_22721_MOESM3_ESM.pdf]

Description of Additional Supplementary Files

Supplementary Movie 1: Curve collapsing with critical exponent $\beta = 5$ and varying $\overbar{\nu}_{alpha}$ for the positively correlated case close to $\alpha = 1$. See Supplementary Note 6 for further details.

Supplementary Movie 2: Curve collapsing with critical exponent $\beta = 16$ and varying $\overbar{\nu}_{alpha}$ for the positively correlated case close to $\alpha = 1$. See Supplementary Note 6 for further details.
